# Mixed-ownership reform and factor misallocation: Evidence from China

**DOI:** 10.1371/journal.pone.0301034

**Published:** 2024-04-16

**Authors:** Ping Peng, Xingwang Zhu

**Affiliations:** 1 School of Economics, Jinan University, Guangzhou, Guangdong, China; 2 Zhejiang Province Key Think Tank: Institute of Ecological Civilization, Zhejiang A&F University, Hangzhou, Zhejiang, China; 3 College of Economics and Management, Zhejiang A&F University, Hangzhou, Zhejiang, China; Tianjin University, CHINA

## Abstract

An enterprise’s ownership structure is crucial for factor allocation efficiency. We used Chinese firm-level data to investigate whether changes in state-owned enterprise ownership structure contribute to resource misallocation, leading to high-quality economic development. We found a U-shaped relationship between non-state shareholding and state-owned enterprises’ resource allocation efficiency. An optimal range exists for non-state shareholding. When the shareholding of non-state shareholders reaches 10%–20%, the efficiency of resource allocation is at its highest. Additional research has revealed that mixed shareholding has varying impacts on resource allocation, displaying substantial heterogeneity. These insights offer valuable guidance for future mixed-ownership reforms and serve as a practical reference for economic reforms in other nations, particularly developing countries.

## Introduction

Some countries are wealthy, whereas others are relatively poor [1]. The reasons for this disparity are many, of which a critical one is the large difference in countries’ total factor productivity (TFP) [2]. Researchers have identified various factors that lead to these differences, and their misallocation has hindered the development of productive forces, particularly in developing countries. Since market reforms began in 1979, China has undergone tremendous economic transformation [3]. The China Report on the Work of the Government 2023 reports that the gross domestic product (GDP) increased to 121 trillion yuan in 2022, with an average annual growth rate of 5.2% in five years. According to the World Bank, China has become the second-largest economy in the world since 2010 [4]. As Linnenluecke et al. (2020) [5]note, topics related to China have received considerable attention in the practical field and in the academic community. After years of economic reform and development, China has developed a unique market system. Research based on the Chinese system can provide perspectives on market development issues and solutions from a different angle than the relevant experiences of other countries—especially developed, Western ones [6]. An important reason for TFP loss is the misallocation of factor markets, which affects economic development. Tu and Xiao (2005) [7]found that enterprises’ resource allocation efficiency has little effect on TFP growth; however, many researchers believe that it is the main contributing factor [8–10]. Regions with developed market economies have higher resource allocation efficiency [11]. According to the aggregate production function (Y = AF(K,L)), the capital market (K) and labor market (L) are directly related to GDP and bring about economic growth; therefore, attention should be paid to misallocation in these two markets [12]. Total factor productivity is positively correlated with labor quality and human capital investment, as well as with high-quality products and market demand. The impact of income gap on the inverted U-shaped total factor productivity and its mechanisms: Evidence from transnational-level analysis [13]. Systems such as household registration and rural land property rights lead to the segmentation of the labor factor market, which leads to a serious mismatch between urban and rural labor [14, 15]. At the same time, capital misallocation among industries has occurred due to financial market constraints and monopoly [16, 17]. The resource allocation between heterogeneous enterprises is a key determinant of significant productivity differences among countries [18]. The process of China’s economic growth and development is essentially uneven [19–21], and resource allocation efficiency in the Chinese economy could be improved [22]. An effective way to achieve high-quality economic development in China is to improve the efficiency of factor resource allocation. The mixed-ownership reform of state-owned enterprises (SOEs) is a special SOE reform method proposed to adapt to China’s gradual economic system reform and a micro-implementation form of the latter [23]. Whether the mixed-ownership reform of SOEs can improve resource allocation efficiency has become a topic of concern in the academic community. Research has found that the proportion of non-state-owned capital has a positive promoting effect on enterprise performance, driving the development of downstream private economy, achieving “national progress”, and becoming a way of industrial layout [24, 25]. Moreover, government intervention tends to deviate SOEs from their efficiency goals, and mixed-ownership reforms can reduce efficiency losses caused by biased policies and promote TFP [26, 27]. However, few studies have examined the impact of ownership on resource misallocation at the enterprise level. The existing literature has generally argued that the resource allocation efficiency of mixed-ownership reform in SOEs is mainly improved to reduce the impact of biased policies and increase the level of profitability and other economic performance. Few studies have directly examined the relationship between the equity depth of mixed-ownership reform and SOEs’ misallocation of resources. This study directly measures resource misallocation at the firm level based on the HK model by considering SOEs in the A-share market of Shanghai and Shenzhen listed companies in 2008–2018 and drawing on Zhang and Deng’s (2020) [28] improved methods. It examines the sum of the shareholding ratios of private and foreign capital among the top 10 shareholders as a proxy index for the depth of SOEs’ mixed-ownership reform. Further, it examines the impact of mixed-ownership reform on SOEs’ misallocation of resources and tests the effectiveness of mixed-ownership reform from an empirical perspective. The contributions of this study are as follows. First, the existing literature has focused on the macro level and used indirect substitution perspectives to study the degree of resource misallocation between enterprises, such as the degree of TFP dispersion [11, 29, 30], TFP decomposition to obtain the industry’s resource allocation efficiency [31], and the HK model using TFPR variance [32]. In contrast, this study directly uses resource misallocation as the entry point; it explores the impact of SOEs’ resource misallocation from the perspective of mixed-ownership reform, enriching the related literature. Second, it explores how non-state-owned shareholders, which reflect market power, play a role in resource misallocation, and it is a useful supplement to the literature on the factors affecting corporate resource misallocation. Third, it discovers a U-shaped relationship between the depth of mixed ownership and degree of resource misallocation in SOEs, thus expanding the research field of mixed-ownership reform. It explores the economic consequences of mixed-ownership reform, advancing the empirical study of non-state shareholders’ shareholding and resource misallocation. Additionally, this study serves as an important decision-making reference for further deepening the reform of SOEs. Studying the laws of the socialist market economy not only deepens our understanding but also fosters the development and innovation of state-owned enterprises. It optimizes resource allocation, enhances economic and social benefits, and facilitates the adjustment and transformation of China’s economic structure. Other developing countries can learn from the problems and solutions in the Chinese market and develop their own markets accordingly.

## Literature review

### Resource allocation efficiency and misallocation

Under the assumption of an efficient market, factors can flow freely to achieve Pareto optimality. In reality, due to factors such as unsound market mechanisms, the Pareto optimal state cannot be achieved: this is called resource misallocation or low resource allocation efficiency, and it manifests in the misallocation of resources within and among enterprises. In Portugal, the misallocation within the industry nearly doubled between 1996 and 2011. Deteriorating allocative efficiency can reduce annual GDP growth by approximately 1.3 percentage points. This may have led to poor economic performance in some southern and peripheral European countries and to a crisis in the eurozone [33]. Oberfield (2013) [34] found that the decrease in capital utilization rate accounts for approximately 25–50% of TFP decline using establishment data from the Chilean manufacturing census.

### Property rights structure and resource allocation efficiency

The average marginal return on capital product differs significantly among firms with different types of ownership; this is typical of China’s economic development [35–37]. Market distortions restrict the free flow of factors of production, resulting in a serious misallocation of capital and labor, which ultimately reduces the output level of China’s manufacturing industry [38]. SOEs are the key to improving the efficiency of resource allocation in China [39]. A mixed-ownership economy can promote the efficiency of SOEs’ resource allocation [40]. With the advancement of reform, the entry of non-state-owned capital into SOEs has brought about changes in the structure of property rights and in operating and management methods [41]. As the proportion of non-state-owned economy increases, non-state-owned shareholders pay more attention to improving the profitability and operating efficiency of SOEs than state-owned shareholders; thus, the efficiency of social labor and capital production is improved [42, 43].

### Effects of mixed-ownership reform in state-owned enterprises

Mixed-ownership reform is a special way of reforming SOEs proposed to adapt to China’s progressive economic system reform and a micro-realization form of the latter. Mixed-ownership enterprises are superior and should be vigorously developed as new growth points for economic development [44]. Government intervention can cause SOEs to deviate from their efficiency goals, and firms with reformed property ownership are more efficient [27]. It is only when the non-state economy enters SOEs, which assume the role of “shareholders”, that private property rights form an effective incentive mechanism of interest and operator selection. The problem of SOEs’ inefficiency can only be solved fundamentally [45]. China’s economic growth is partly explained when resources are shifted from inefficient state-owned sectors to efficient, non-state-owned sectors [8]. With the reform development and opening up, mixed-ownership reform has become an important means to SOE reform, while private enterprises develop and grow.

### Shareholding of non-state-owned shares and performance of mixed-ownership reform

Owing to SOEs’ special nature and status, it is necessary to comprehensively consider the effects of mixed-ownership reform on corporate performance and social responsibility. The entry of non-state-owned capital affects policy decisions and behaviors to a certain extent. In theory, if private and state-owned shares’ shareholding ratio can reach the optimal mixing ratio, welfare may be maximized [46]. An inverted U relationship exists between ownership concentration, ownership balance of SOEs, and corporate performance and between the proportion of non-state-owned shareholders and performance of SOEs [47]. Biased policies lead to distortions in the input of production factors in SOEs; however, mixed-ownership reform can reduce the efficiency loss of SOEs, and some additional factors will affect the optimal shareholding ratio of mixed-ownership reform [48]. Affected by the negative externalities of production, the cost of SOEs, product differentiation, and the number of private enterprises in the industry will reduce the optimal equity ratio of mixed-ownership reform. A literature review revealed several documents on the impact of mixed-ownership reform on SOEs, and studies that have examined the role of mixed-ownership reform from the perspective of production efficiency (business performance) and technical efficiency. The literature on the impact of mixied-ownership reform from the factors of production’s input efficiency is still scarce and mostly concentrated at the industry level [47, 49]. Even fewer studies have quantified SOEs’ resource allocation efficiency from the perspective of micro-enterprises and studied the impact of blending. Therefore, the existing literature has mainly addressed the impact of blending reform on resource mismatch in SOEs. This study begins with the degree of resource mismatch and establishes a research framework to examine the effect of blending on resource allocation efficiency.

## Theoretical analysis and hypothesis

**Fig 1:**
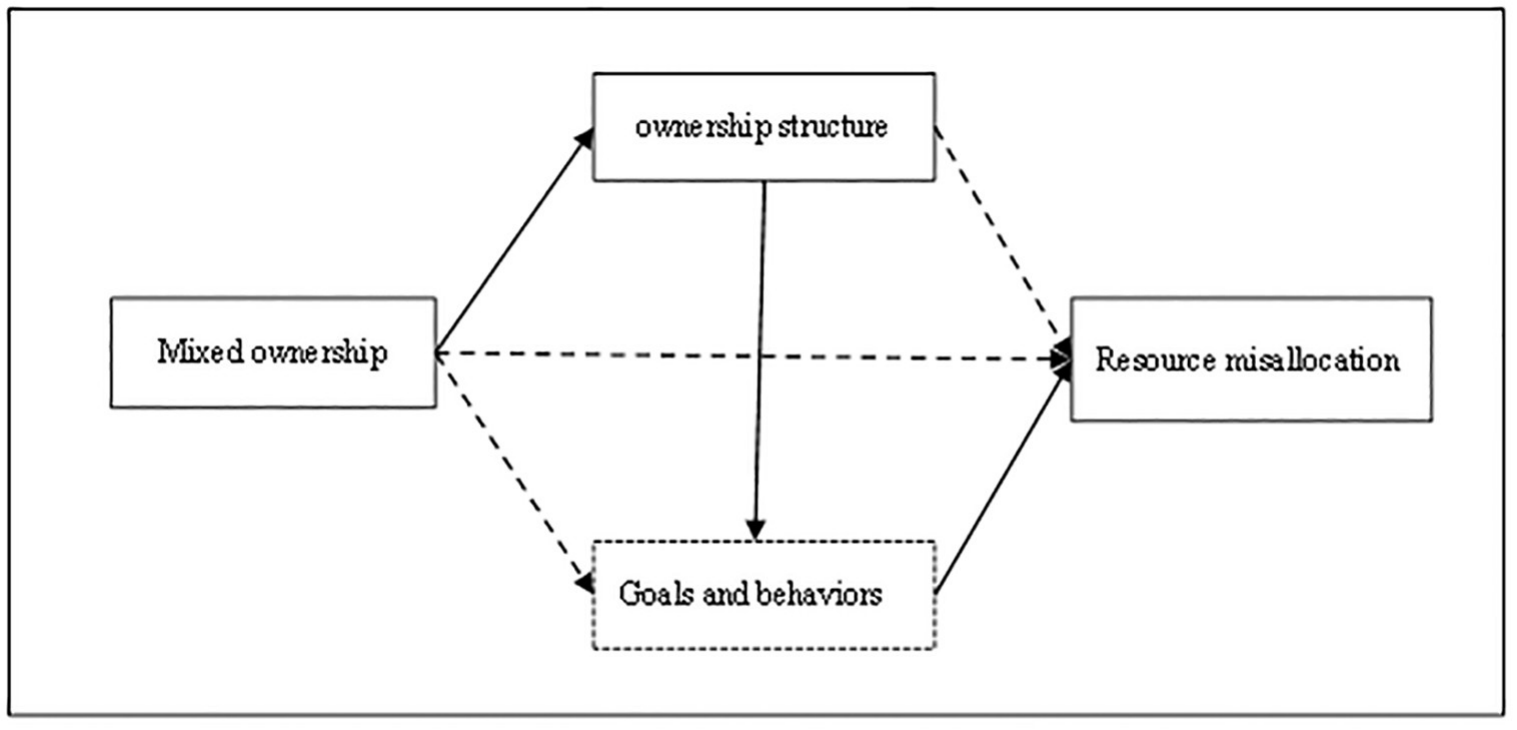
Logical framework diagram.

Modern property rights theory posits that the property rights system is key to promoting economic development, and social resources allocation and economic efficiency vary under different property rights systems. Governments should promote economic growth and social welfare by reforming and improving the existing property rights system. A critical aspect of the mixed-ownership reform of SOEs is property rights reform, which involves the introduction of non-state-owned equity into SOEs. Improving the efficiency of factor resource allocation is an effective way to improve China’s TFP and achieve high-quality economic development. Does the change in ownership structure brought about by the mixed-ownership reform impact SOEs’ resource allocation efficiency, and what is the logic behind it? This study proposes a research framework for mixed-ownership reform: ownership structure—goal and behavior—resource mismatch (Fig 1).

## Research design

### Model setting

To test H1 and verify the U-shaped relationship between the depth of mixed shareholding reform and the degree of resource mismatch among SOEs, we establish the following measurement models:

$$\begin{array}{c} \;MA_{it}=\beta_{0}+\beta_{1}\times nonshr_{it}\;+\beta_{2}\times nonshr_{it}^{2}+\beta_{3}\times Z_{it}+\sum i+\sum t+\varepsilon_{it} \end{array}$$ (1)

In model (1), the explained variable *MA*_*it*_ is the enterprise’s degree of resource mismatch in the first year, calculated by referring to Zhang and Deng (2020) [28]. *MA*_*it*_ denotes the degree of deviation between the actual output scale and the optimal output scale due to the distortion of factor allocation. The larger the deviation, the more serious the distortion, the lower the efficiency of resource allocation, and the higher the degree of mismatch. The core explanatory variables include *nonshr*_*it*_ and $nonshr_{it}^{2}$, which represent the equity depth of enterprise i in year t and the square term of equity depth. Equity penetration refers to the proportion of non-state-owned shares held, which is measured by the sum of the proportion of private and foreign shares held by the top 10 shareholders. *Z*_*it*_ is the model’s control variable. Definition of the variables and description of the indicators can be found in Table 1.

**Table 1:** Definition of the variables and description of the indicators.

Type of variable	Variable Symbol	Variable Name	Formula for variables
Explained variables	*MA*	Level of resource misallocation	Overall level of resource misallocation, as measured by the authors [8, 28]
Explanatory variables	*nonshr*	Shareholding of non-state shareholders	Sum of private and foreign shareholdings among the top 10 shareholders
	*nonshr* ^2^	Squared term of shareholding of non-state shareholders	Squared term of shareholding of non-state shareholders
Mechanism variables	*τ*_*K*_	Capital input distortion	Degree of distortion of capital factor inputs, as measured by the authors [8, 28]
	*τ*_*L*_	Labor input distortion	Degree of distortion of labor factor inputs, as measured by the authors [8, 28]
	*R*_*e*_	Cost of Capital	Required rate of return on capital, calculated according to the capital asset pricing model (CAPM)
Control variables	*age*	Firm age	Logarithm after subtracting the year of listing plus 1 from the observation year
	*apc*	Assets per capita	Logarithm of fixed assets per capita at the end of the year
	*lev*	Capital structure	Total liabilities / total assets
	*growth*	Revenue growth rate	(Yearend revenue—previous year’s yearend revenue)/ previous year’s yearend revenue
	*gshr*	Shareholding of financial shareholders	Shareholding of financial shareholders among the top 10 shareholders
	*excushr*	Shareholding of executive shareholders	Number of shares held by executives divided by the total number of common shares multiplied by 1000
	*hhi*	Ownership concentration	Herfindahl index of top 10 shareholders’ shareholdings
	*gdp*_*per*	City-level controls for city GDP per capita	Logarithm of the per capita GDP level of the city where the enterprise is located
	*pop*	City population	Logarithm of the population of the city where the enterprise is located in the current year

### Data source

With market-oriented reforms in China, the reform of non-tradable shares was officially launched in 2005 and completed by the end of 2007 for listed companies. Before then, it had been more difficult for non-state capital firms to enter state-owned listed companies [50]. Thus, the reform of non-tradable shares has had an important impact on SOEs’ equity structure. Therefore, our main analysis focuses on A-share listed SOEs in Shanghai and Shenzhen from 2008 to 2018. To facilitate the observation of the mixed-ownership reform’s effect, data from at least 2 years after the implementation of the mixed-ownership reform were retained, and companies listed in 2016 and before were selected. According to the actual control standard, enterprises were classified into SOEs, private enterprises, foreign-funded enterprises, and other enterprises. To maintain the robustness and reliability of the empirical results, this study selected samples that have been SOEs from 2008 or whose year of listing is 2018 and made the following screening treatments: (1) excluding the samples of financial enterprises; (2) excluding the samples with missing or abnormal data; and (3) excluding the samples of ST listed companies during the period. At the same time, extreme values were also treated as follows: (1) excluding 1% of observations before and after the key variables to eliminate the influence of extreme values; and (2) excluding 1% of observations before and after the first calculation of capital factor input distortion and labor factor input distortion, and then performing subsequent calculations. Finally, 6759 annual observation samples from 732 companies were obtained.

### Definition of variables

Dependent variable: Level of resource misallocation. As one of the driving forces of economic growth, resource allocation efficiency refers to how to promote the flow of resources from areas with low marginal productivity to areas with high marginal productivity, based on established resources and technology, to more effectively and reasonably utilize resources and maximize total social value. A single-factor input, as reflected at the micro-enterprise level, refers to the connection between a factor input’s marginal return and marginal cost. When the factors are assigned optimally, marginal revenue equals marginal cost. When marginal income exceeds marginal cost, the factor input is distorted to be positive and insufficient; when marginal income is less than marginal cost, the factor input is distorted to be negative, resulting in excessive factor input. Drawing on the framework for measuring the degree of resource misallocation at the firm level proposed by Zhang and Deng (2020) [28], we innovatively measure the distortion of factor inputs and the degree of resource misallocation of listed SOEs. The greater the degree of resource mismatch, the lower the efficiency of resource allocation. The specific calculation formula and model are as follows. Assuming that each differentiated product is produced by two production factors, capital and labor, invested by a monopolistic enterprise, the production function is in the C-D form:

$$\begin{array}{c} \;Y_{\text{si}}=A_{si}K_{si}^{\alpha_{s}}L_{si}^{\beta_{s}} \end{array}$$ (2)

Among them, *A*_*si*_ is the TFP level of enterprise. *α*_*s*_ represents the capital elasticity of industry s. *β*_*s*_ represents the labor elasticity of industry s, and $\alpha_{s}+\beta_{s}=1.\tau_{K_{si}}$ and $\tau_{L_{si}}$ represent capital distortion and labor distortion, respectively. The profit function of monopolistic competitors is expressed as Eq 3:

$$\begin{array}{c} \;\;\pi_{si}=P_{si}Y_{si}-\left(1+\tau_{L_{si}}\right)\omega L_{si}-\left(1+\tau_{K_{si}}\right)RK_{si} \end{array}$$ (3)

where P denotes the product price. R is the capital price of the enterprise, and *ω* is the labor price for the enterprise. According to the first-order condition for maximizing profit, we obtain Eq 4:

$$\begin{array}{c} \;\frac{K_{si}}{L_{si}}=\frac{\left(1+\tau_{L_{si}}\right)\omega \alpha_{s}}{\left(1+\tau_{K_{si}}\right)R\beta_{s}} \end{array}$$ (4)

According to marginal revenue equal to marginal cost,

$$\begin{array}{c} \;MRPK_{si}\equiv \alpha_{s}\frac{\sigma -1}{\sigma }\frac{P_{si}Y_{si}}{K_{si}}=\left(1+\tau_{K_{si}}\right)R \end{array}$$ (5)

$$\begin{array}{c} \;MRPL_{si}\equiv \beta_{s}\frac{\sigma -1}{\sigma }\frac{P_{si}Y_{si}}{L_{si}}=\left(1+\tau_{L_{si}}\right)\omega \end{array}$$ (6)

Therefore, it is possible to determine the distortion of capital and labor input factors faced by enterprises:

$$\begin{array}{c} \;\tau_{K_{si}}=\alpha_{s}\frac{\sigma -1}{\sigma }\frac{P_{si}Y_{si}}{RK_{si}}-1 \end{array}$$ (7)

$$\begin{array}{c} \;\tau_{L_{si}}=\beta_{s}\frac{\sigma -1}{\sigma }\frac{P_{si}Y_{si}}{\omega L_{si}}-1 \end{array}$$ (8)

Among them, *σ* representing the elasticity of substitution between products. The substitution elasticity in competitive manufacturing (*σ*) is generally 3–10 [51, 52]. This study draws inspiration from Zhang and Deng (2020) [28] and conservatively estimates that *σ* is 3. The enterprise’s actual output scale and optimal output scale when distortion is considered are shown in Eqs 9 and 10, respectively.

$$\begin{array}{c} \;Y_{K_{si}}=Y_{si}\times {\left(1+\tau_{K_{si}}\right)}^{\sigma \alpha_{s}} \end{array}$$ (9)

$$\begin{array}{c} \;Y_{L_{si}}=Y_{si}\times {\left(1+\tau_{L_{si}}\right)}^{\sigma \beta_{s}} \end{array}$$ (10)

$$\begin{array}{c} \;Y_{E_{si}}=Y_{L_{si}}\times {\left(1+\tau_{K_{si}}\right)}^{\sigma \alpha_{s}}\times {\left(1+\tau_{L_{si}}\right)}^{\sigma \beta_{s}} \end{array}$$ (11)

The formula for resource misallocation at the enterprise level (*MA*_*si*_) is

$$\begin{array}{c} \;MA_{si}=\frac{Y_{E_{si}}}{Y_{si}}-1={\left(1+\tau_{K_{si}}\right)}^{\sigma \alpha }{\left(1+\tau_{L_{si}}\right)}^{\sigma \beta }-1 \end{array}$$ (12)

*MA*_*si*_ represents the degree to which a company’s actual output scale deviates from the optimal output scale due to distorted factor allocation. When the degree of deviation is greater, the distortion faced by the enterprise is more severe; the efficiency of the enterprise’s factor resource allocation is lower, and the degree of resource mismatch is higher. The measurement process requires the enterprise’s industrial value added in the current year. In 2008, the National Bureau of Statistics (NBS) reformed the measurement method for industrial value added; however, obtaining industrial value-added data directly at the enterprise level has not been possible. The methods for calculating industrial value added are two: the production method, which is the total industrial output—industrial intermediate inputs + value-added tax payable; and the income method, which is calculated from the perspective of income, based on the share of income due to production factors in the production process, with specific component items such as depreciation of fixed assets, labor compensation, net production tax, and operating surplus. In this study, the income method is used to manually compile and calculate the sample enterprises’ industrial value added and perform subsequent index calculations and empirical analysis.

Industrial added value = depreciation of fixed assets + employee compensation + net production tax + operating surplus. Existing literature and statistical methods have shown a consensus regarding the depreciation of fixed assets and employee compensation. Depreciation of fixed assets refers to the depreciation amount of the enterprise’s fixed assets for the current year. Employee compensation refers to the cash paid to and for employees in the current year. The net production tax and operating surplus are calculated using the statistical methods published by the local statistical bureau and obtained from the WIND database. Net production tax = taxes payable—government subsidies. Operating surplus = operating profit + union expenses * 0.4 + management fees paid in management expenses—interest income * 0.06 + interest expenses * 0.06—fair value change benefits—investment income—asset disposal income.

Furthermore, the degrees of enterprises’ capital input distortion, labor input distortion, and resource misallocation are compared with those measured by Zhang and Deng (2020) [28] using a database of Chinese industrial enterprises in 1997–2007 (Table 2, with the results of Zhang and Deng (2020) [28] in parentheses). By comparison, the degrees of factor distortion and resource misallocation measured in this study are in the same direction, and the overall degree of distortion or misallocation is smaller. To a certain extent, this corroborates the accuracy of the previous manual calculation of the industrial value added. The calculation of deflation is illustrated by setting 2008 as the base period and using the fixed asset investment price index for each year to deflate net fixed assets, the industrial ex-factory price index to deflate industrial value added, and the consumer price index to deflate total wages, with data obtained from the official website of the National Bureau of Statistics (http://www.stats.gov.cn).

**Table 2:** Definition of the variables and description of the indicators.

Variable name	Number of samples	mean value	Standard deviation
Capital input distortion	6759(1099000)	1.943(3.426)	3.906(4.061)
Labor input distortion	6759(1099000)	-0.060(-0.181)	0.614(0.816)
Level of resource misallocation	6759(1099000)	7.663(13.91)	25.244(35.11)

Data from authors’ calculations [28]

Independent variable: Shareholding of non-state shareholders. Referring to Hao and Gong (2017) [53] on the division of the nature of shareholding, the company’s top 10 shareholders were classified into four categories: “state-owned shareholders”, “private shareholders”, “foreign shareholders”, and “financial shareholders”. We used documents of the top 10 shareholders of the Guotai An database (CSMAR) to obtain information on the top 10 shareholders of the sample enterprises from 2008 to 2018. When the shareholders are marked as H-shares—shareholders of overseas legal persons—the actual controller may be state-owned. When the shareholder is a domestic legal person, the actual controller may be state-owned, private, or foreign-owned. Therefore, in accordance with the principle of actual controller and controlling shareholding, the nature of legal person shareholders among the top 10 shareholders was manually verified one by one through the enterprise’s annual report and the websites of Tianyancha and Enterprise Inspection. For enterprises whose nature is unclear, the authenticity and availability of data are guaranteed by means of the enterprise’s official website, government news information, the official website of the local State-owned Assets Supervision and Administration Commission, and the directory of local SOEs. Due to the significant difference between the nature of financial shareholders and other shareholders, referring to the practice of Hao and Gong (2017) [53], “financial shareholders” were separated from “state-owned shareholders” and “private shareholders”, and the top 10 shareholders in the sample of SOEs screened by listed companies in 2008–2018 totaled 81766 (including duplicate shareholders in different years). Table 3 presents the shareholders’ specific groupings.

**Table 3:** Overview of shareholder groups in state-owned enterprises.

Nature of shareholders	Definition	Average shareholding	Subtotal
State-owned shareholders	All levels of government departments (such as the Ministry of Finance and the State-owned Assets Supervision and Administration Commission), state-owned enterprises, and the four major asset management companies and their wholly owned subsidiaries, excluding financial shareholders	14.548%	25801
Private shareholders	Non-state corporate entities and domestic natural persons Excluding financial shareholders	1.595%	25000
Financial shareholders	National social security fund, securities investment fund, insurance investment account, trust account, bank fund account, etc.	1.197%	27916
Foreign shareholders	H shares, foreign corporate entities, and foreign natural persons	7.225%	3049

Mixed-ownership reform is measured by the presence of private or foreign shareholders among a company’s top 10 shareholders. Specifically, the shareholdings of non-state shareholders (*nonshr*_*it*_) and shareholding balance (*mor*_*it*_) are used to represent the degree of equity mix reform; equity depth is used as benchmark regression, and equity balance is used as robustness test. The shareholding of non-state shareholders (*nonshr*_*it*_) is the sum of private and foreign shareholdings among the top 10 shareholders, and the greater the equity depth, the deeper the hybridization. Shareholding balance (*mor*_*it*_) is the difference between the sum of private and foreign shareholdings among the top 10 shareholders minus the proportion of state-owned shares; the greater the degree of shareholding balance, the greater the degree of mixed-ownership reform [47].

Control variable. The following variables are used as control variables, together with firm and year taken as control variables: firm age (*age*), assets per capita (*apc*), capital structure (*lev*), sales revenue growth rate (*growth*), shareholding of financial shareholders (*gshr*), shareholding of executive shareholders (*excushr*), and ownership concentration (*hhi*) at the firm level. City-level controls for city GDP per capita (*gdp*_*per*) and population (*pop*). Individual fixed effects (∑*i*) and year fixed effects (∑*t*) are also included to reflect the effect of individual and time-unobservable characteristics on the degree of resource misallocation. The random disturbance term (*σ*_*it*_) contains factors that are not controlled for in the model but impact the degree of resource mismatch. Table 1 lists the specific variable names, meanings, and calculation methods.

### Descriptive statistics of variables

The study period is 2008–2018, which provides a good data-analysis basis for SOEs’ mixed-ownership reform structure in listed companies. The overall number and proportion of enterprises in the SOE sample that underwent mixed-ownership reform show an upward trend. Among these, 79% of the SOEs in the sample implemented mixed-ownership reform in 2008. Overall, the average depth of mixed-ownership reform among SOEs shows an annual upward trend not exceeding 8%. With the promotion of such reform, equity depth gradually increases, and the degree of resource mismatch of SOEs shows a decreasing trend followed by an increasing trend (Table 4). When the depth of the mixed-ownership reform is between 10% and 20%, the degree of resource mismatch reaches its lowest value, and the resource allocation efficiency of SOEs is the highest. The sample size of equity depth below 10% reaches 5289, accounting for 78.25% of the total sample of SOEs. This indicates that, from the perspective of optimizing resource allocation efficiency, most SOEs have room to further deepen mixed-ownership reform. Ma et al. (2015) [47] highlight an inverted U relationship between the depth of SOEs’ mixed-ownership reform and firm performance. When resource allocation efficiency is high, business performance is good; therefore, some rationality exists between the two.

**Table 4:** Comparison of resource misallocation of companies with different shareholdings of non-state shareholders.

Shareholding of non-state shareholders	Capital input distortion	Labor input distortion	Level of resource misallocation	Number of samples
Less than 10%	1.98278	-0.0856466	7.697366	5289
10%–20%	1.64104	-0.0616972	3.980407	743
20%–30%	1.90773	0.1374892	10.04432	390
30%–40%	1.574067	0.1256903	12.04493	249
40%–50%	3.096299	0.1387299	13.67933	49
Greater than 50%	3.643394	0.0285849	13.73347	39
Total	1.943481	-0.0600674	7.66255	6759

Data from empirical results.

Table 5 presents the descriptive statistical analysis of the main variables. The mean value of equity depth is 6.7%, which is lower than the 10% of Cai et al. (2018) [50]; this may be because the non-state shareholder holdings in this study are the top 10 shareholders and do not include financial shareholder holdings. From the perspective of enterprises’ factor input distortion, the mean value of capital distortion is 1.943, and that of labor distortion is -0.06. The mean value of capital input distortion is greater than 0, which means that the marginal return of the enterprise’s capital input is much higher than the marginal cost of capital, and the quantity of capital input is seriously insufficient. The mean value of labor input distortion is less than 0, which means that the marginal return of the enterprise’s labor input is lower than the marginal cost of labor, and the labor factor input is excessive.

**Table 5:** Descriptive statistics of main variables.

Main variables	N	Mean	Sd	Min	Max
*τ*_*K*_	6759	1.943	3.906	-0.974	35.692
*τ*_*L*_	6759	-0.06	0.614	-0.975	3.142
*MA*	6759	7.663	25.244	0.001	305.983
*nonshr*	6759	0.067	0.1	0	0.738
*nonshr* ^2^	6759	0.014	0.039	0	0.544
*age*	6759	2.413	0.678	0	3.367
*apc*	6759	14.477	0.997	11.788	19.712
*lev*	6759	0.49	0.191	0.01	0.964
*growth*	6759	0.181	0.978	-0.862	56.174
*gshr*	6759	0.041	0.047	0	0.503
*excushr*	6759	0.003	0.015	0	0.328
*hhi*	6759	0.2	0.13	0.003	0.76
*gdp*_*per*	1809	10.771	1.018	1.656	13.321
*pop*	1809	5.702	1.022	2.066	8.133
*mkt*	341	6.091	2.052	-0.23	10

The power of state-owned capital does not lie in the current capital stock but in how much capital it can control [54]. The maximum value of equity depth is 73.8%, which indicates that in the process of SOEs’ mixed-ownership reform, state-owned capital, as the actual controller, reflects that the control of state-owned capital has become stronger, although private and foreign shareholdings, among the top 10 shareholders, show absolute dominance. The proportion of enterprises that have undergone mixed-ownership reform in the sample time period of listed companies in the SOE reform process reaches more than 80%. Existing studies on the efficiency of SOEs’ mixed-ownership reform have focused on economic performance (return on assets and net market ratio). Conversely, this study starts with the concept of resource mismatch, focuses on micro subjects, and manually measures the degree of resource mismatch among SOEs. The relationship between actual and optimal output size is compared when the inputs are given. This is more directly indicative of the extent to which firms exploit the potential productivity of resources than of economic performance.

## Empirical results

### Basic regression analysis

To test the hypotheses, Model (1) is regressed using the data of SOEs listed in Shanghai and Shenzhen in 2008–2018. The results are shown in Column (1) of Table 6, where the coefficient of the non-state shareholding ratio’s quadratic term is significantly positive and that of the primary term is significantly negative; this indicates a U-shaped relationship between the non-state shareholding ratio and SOEs’ resource allocation efficiency.

**Table 6:** Basic regression results and formation mechanisms.

Variables	(1)	(2)	(3)	(4)	(5)
	*MA*	*τ*_*K*_	*τ*_*L*_	*R*_*e*_	*τ*_*L*_
*nonshr*	-14.51*	-3.214***	0.269	-0.0115***	0.243**
	-8.421	-1.208	-0.199	-0.00393	-0.102
*nonshr* ^2^	43.40**	8.890***	-0.0681		
	-19.29	-2.703	-0.445		
*age*	-3.965***	-1.333***	-0.101***	-0.00206**	-0.101***
	-0.803	-0.14	-0.023	-0.000886	-0.023
*apc*	4.580***	0.130*	0.153***	-7.41E-05	0.153***
	-0.464	-0.0725	-0.0119	-0.00046	-0.0119
*lev*	0.0847	-0.0963	-0.595***	-0.00196	-0.595***
	-2.257	-0.343	-0.0565	-0.00217	-0.0565
*growth*	0.785***	0.118***	0.0592***	0.000406**	0.0593***
	-0.214	-0.0289	-0.00477	-0.000184	-0.00477
*gshr*	14.49**	2.029**	0.843***	-0.0344***	0.841***
	-5.729	-0.791	-0.13	-0.00499	-0.13
*excushr*	88.06***	13.73***	-0.601	-0.0731**	-0.609
	-29.03	-4.749	-0.783	-0.0301	-0.782
*hhi*	-1.091	1.715**	0.301***	-0.00269	0.299***
	-4.098	-0.671	-0.111	-0.00422	-0.11
*gdp*_*per*	0.0522	-0.400***	-0.0414*	0.000861	-0.0414*
	-0.702	-0.137	-0.0227	-0.000872	-0.0227
*pop*	0.761	1.303***	0.0244	-0.00351*	0.0243
	-0.857	-0.324	-0.0534	-0.00206	-0.0534
*Year*	Controlled	Controlled	Controlled	Controlled	Controlled
*Firm*	Controlled	Controlled	Controlled	Controlled	Controlled
_*cons*	-58.164***	-2.428	-1.594***	0.141***	-1.592***
	-10.035	-2.255	-0.372	-0.0143	-0.371
*Adj* − *R*^2^	0.034	0.071	0.101	0.462	0.101
*N*	6759	6759	6759	6758	6758

T-values are in parentheses;

***, **, and * indicate statistical significance at the 1%, 5%, and 10% levels, respectively.

The critical value is calculated to be near 17.95% and passes the U-shaped relationship test at the 5% level. The extreme value point 17.95% is within the data range, and the slope in the result is first negative and then positive in the interval. Combined with the coefficients in the benchmark regression model, this study suggests a U-shaped relationship between the degree of equity depth and that of resource misallocation among SOEs. That is, when equity depth increases from 0 to 17.95%, SOEs’ resource mismatch is alleviated with the increase of non-state shares’ shareholding. When equity depth is 17.95%, SOEs’ resource mismatch is mitigated to the greatest extent. When equity depth exceeds 17.95%, the increase of non-state shareholding leads to the deterioration of resource mismatch in SOEs. In other words, the optimal range of equity depth is 10%–20%, which is consistent with the data above. The regressions show that, overall, a U-shaped relationship exists between equity depth in mixed-ownership reform and the degree of resource mismatch in SOEs. Non-state shareholders’ shareholding (i.e., the proportion of non-state shares introduced) is not necessarily better, and complete nationalization does not necessarily result in the highest efficiency of SOEs’ resource allocation. The best approach is to reduce state-owned shares and introduce non-state-owned shareholders, forming a balanced structure between state-owned and non-state-owned shareholders, which is conducive to improving enterprises’ resource allocation efficiency. With an increase in non-state shareholding, the degree of SOEs’ resource misallocation is mitigated; however, when non-state shareholding reaches a critical value, the degree of SOEs’ resource misallocation worsens due to the increase in non-state shareholding.

### Mechanism analysis

As capital input distortion and labor input distortion are used as factors to calculate the degree of resource misallocation, the explanatory variables are replaced with capital input distortion and labor input distortion, respectively, and regression is applied to Model (1). The results, as shown in Columns (2) and (3) of Table 6, show a significant U-shaped relationship between shareholding by non-state shareholders and capital input distortion, which passes the subsequent U test. SOEs’ capital input distortion reaches the minimum when non-state shareholders’ shareholding is 18%; that is, the optimal interval of non-state shareholders’ shareholding is 10%–20%. The inverse U-shaped relationship between non-state shareholders’ shareholding and labor input distortion is not significant.

This study argues that the formation of the U-shaped relationship also stems from the combined effect of reduction in the cost of financing (the cost of capital input) and the loss of labor input. First, the capital asset pricing model (CAPM) is used to calculate the cost of capital (*R*_*e*_) for the sample SOEs from 2008 to 2018 using data from annual reports of listed companies (WIND). The cost of capital, as the intrinsic rate of return on capital required by external investors, is used to measure the ease of financing; a higher value indicates that raising capital is more difficult. The CAPM model is used in the calculation process. Based on the principle of risk compensation, the cost of capital is equal to the risk-free reward plus the risk premium.

$$\begin{array}{c} \;R_{e}=R_{f}+\beta \left(R_{m}-R_{f}\right)\; \end{array}$$ (13)

Among them, the risk-free payoff rate *R*_*f*_ selected from the compound interest rate of treasury bonds issued annually for more than 5 years; the market risk premium (*R*_*m*_-*R*_*f*_) is substituted using Damodaran’s estimation data on the risk premium of the Chinese stock market, and *β* is the market risk coefficient, which is sourced from the RESET database. The mean cost of capital is 12.49%; the median is 12.38%; the minimum is 2.25%, and the maximum is 33.14%.

A comparative analysis of the impact of non-state shareholders’ shareholding of on capital cost and labor input distortion shows that as the depth of mixed-ownership reform—that is, the proportion of non-state-owned shares—increases, capital cost significantly decreases, and labor input distortion is significantly positive, as shown in Columns (4) and (5) of Table 6. This verifies the U-shaped relationship between non-state shareholders’ shareholding and SOEs’ misallocation of resources. In the early stage of mixed-ownership reform, with the entry of non-state-owned shares, non-state shareholders’ shareholding increased; the cost of absorbing capital for SOEs decreased, and their ability strengthened. At the same time, a significant positive correlation exists between the distortion of labor investment and non-state shareholders’ shareholding, which initially alleviated the phenomenon of excessive labor investment in SOEs and, to some extent, verified the alleviation effect of mixed-ownership reform on the problem of redundancy in newly listed SOEs [55]. The degree of resource misallocation in SOEs improves, forming the first half of the U-shaped relationship. When non-state shareholders’ shareholding reaches the optimal shareholding ratio, the degree of resource misallocation in SOEs is minimized, and the efficiency of resource allocation is the highest. However, when the optimal shareholding amount is exceeded, the reform of non-state shareholders’ shareholding continues to increase. Although the cost of capital decreases, the distortion of labor input worsens, ultimately exacerbating the degree of resource mismatch in SOEs and forming the second half of the U-shaped relationship.

### Robustness analysis

#### Substitution of explanatory variables

Non-state shareholders’ shareholding is replaced by shareholding balance, where shareholding balance = proportion of non-state shareholding—proportion of state shareholding, and the greater the shareholding balance, the deeper the degree of mixed-ownership reform. The regression results are shown in Column (1) of Table 7; an inverted U relationship is observed between shareholding balance and enterprises’ resource allocation efficiency, which is significant at the 5% level and passes the U test with an extreme value point of -39.36%. In other words, resource allocation efficiency improves when the percentage of state-owned shares minus the percentage of non-state-owned shares is less than 39.36%. When the difference between the two is greater than 39.36%, resource misallocation increases. This indicates that the benchmark regression results are robust.

**Table 7:** Robustness tests.

*MA*	(1)	(2)	(3)	(4)
	Substitution of explanatory variables	Heckman two-stage regression	Period 2009–2018	Period 2008–2016
*mor*	-5.631***			
	-2.079			
*mor* ^2^	-7.153**			
	-3.44			
*nonshr*		-17.61*	-15.84*	-21.48**
		-9.355	-9.462	-9.436
*nonshr* ^2^		45.71**	48.50**	61.59***
		-20.34	-21.09	-21.19
*lambda*		41.82***		
		-3.495		
*Controlvariables*	Controlled	Controlled	Controlled	Controlled
*Year*	Controlled	Controlled	Controlled	Controlled
*Firm*	Controlled	Controlled	Controlled	Controlled
_cons	-68.38***	-68.62***	-43.81**	-32.20*
	-6.042	-16.97	-17.94	-17.07
*Adj* − *R*^2^	0.249	0.061	0.029	0.039
*N*	6759	5636	6225	5453

T-values are in parentheses;

***, **, and * indicate statistical significance at the 1%, 5%, and 10% levels, respectively.

#### Exclusion of enterprises’ selection bias

Firms’ production decisions and financial performance may be affected by hybridization or other factors, which may further affect their exit or listing. In other words, a potential self-selection problem in the enterprises’ entry or exit may affect the regression results. To address this issue, a Heckman two-stage regression is used to examine the effect of equity depth on resource misallocation in SOEs. In the first stage, whether mismatching occurs is estimated by taking the logarithm of the firm’s asset size, net asset margin, gearing ratio, employment, and the marketization process of the region where the firm is located. The obtained inverse Mills ratio is added as a control variable in the second-stage regression, as shown in the regression results in Column (2) of Table 7; the quadratic term of non-state shareholders’ shareholding is significantly positive, and the primary term is significantly negative, passing the U test. The lambda is significant at the 1% level, which indicates a significant U-shaped relationship between equity depth and the degree of SOEs’ resource mismatch. Thus, the basic regression results are robust and reliable.

#### Interval robustness test

Given the impact of the 2008 global financial crisis on the overall economic operations and stock market in China, the selection period starts in 2009 to examine whether a U-shaped relationship exists between non-state shareholders’ shareholding and the degree of SOEs’ resource misallocation. The sample period is shortened, and only the data from 2009–2018 are selected as the sample to retest; the results are not substantially different. The regression results and U test in Table 7 (3) indicate a U-shaped relationship between the shareholding of non-state shareholders and the degree of SOEs’ resource misallocation. The optimal extreme value point—that is, the optimal non-state shareholding ratio—is 16.33%, and the baseline regression results are robust and reliable. Considering that the entry of non-state capital may have a certain postponement-driven effect on the behavior of SOEs’ business objectives and corporate governance, the sample is selected for regression in 2008–2016, and the results are shown in Column (4) of Table 7, where a significant U-shaped relationship exists between non-state shareholders’ shareholding and the degree of SOEs’ resource misallocation. The optimal shareholding is 17.44% by quadratic test, which is within the optimal interval range. The benchmark regression results are verified to be robust and reliable.

## Discussion

### Industry competition level

Boosting resource allocation efficiency through the promotion of market-oriented reforms of various productive factors and the gradual establishment of a rational pricing system is crucial. This would result in substantial enhancements to China’s industrial TFP and a considerable boost in economic growth [56]. Monopolistic industries were divided according to Aharony et al. (2000) [57] and Zhao et al. (2017) [58]. Referring to the Report on the Economic Performance of Central State Enterprises (2012), we divided the SOE sample into monopolistic and competitive industries. Eight industries were selected as monopolistic industries: coal industry, petroleum and petrochemical industry, metallurgical industry, civil engineering and construction industry (mainly referring to railroad, tunnel, port, and other engineering industries), transportation industry (mainly referring to railroad transportation, water transportation, air transportation), electric power industry, telecommunication industry, and other industries (mainly referring to press and publishing industry). Enterprises were competitive industries. As shown in Columns (1) and (2) of Table 8, the regression results indicate that the U-shaped relationship between the shareholding of competitive SOEs’ non-state shareholders and the degree of enterprises’ resource misallocation is significant, and the extreme value point of equity depth lies within the optimal interval. This echoes the SOE classification reform policy and verifies, to a certain extent, that competitive SOEs can improve their resource allocation efficiency by deepening the mixed-ownership reform path.

**Table 8:** Expanded analysis: Enterprise nature and regional differences.

*MA*	(1)	(2)	(3)	(4)	(5)	(6)	(7)
	Monopoly industry	Competitive industry	Capital intensive industry	Labor intensive industry	Eastern region	Central region	Western region
*nonshr*	25.33	-22.72**	-13.20*	-5.887	-23.57*	1.466	-18.07
	-20.97	-9.927	-7.659	-16.84	-13.99	-6.67	-20.28
*nonshr* ^2^	-17.73	54.42**	28.35*	52.53	77.87**	12.62	3.635
	-43.95	-22.44	-17.12	-36.69	-33.7	-14.5	-41.94
*Controlvariables*	Controlled	Controlled	Controlled	Controlled	Controlled	Controlled	Controlled
*Year*	Controlled	Controlled	Controlled	Controlled	Controlled	Controlled	Controlled
*Firm*	Controlled	Controlled	Controlled	Controlled	Controlled	Controlled	Controlled
_*cons*	-228.3***	-51.54***	29.57	-111.5***	-193.0***	-11.5	-10.87
	-82.36	-18.28	-26.82	-29.02	-71.04	-10.06	-126.3
*Adj* − *R*^2^	0.068	0.039	0.028	0.047	0.047	0.051	0.055
*N*	1372	5387	3380	3379	4197	1507	1055

T-values are in parentheses;

***, **, and * indicate statistical significance at the 1%, 5%, and 10% levels, respectively.

### Capital intensity

Introducing diversified ownership interests can resolve internal governance issues in SOEs and improve their efficiency and competitiveness [59]. Next, this study explains why capital input distortion in the previous evidence mechanism analysis has a significant U-shaped relationship with mixed equity depth, while labor input distortion is insignificant from the capital intensity perspective. Capital intensity is measured in terms of fixed assets per capita. When a firm’s fixed assets per capita is higher than the median fixed assets per capita of all firms, it is considered highly capital intensive. Otherwise, it has a low capital intensity, that is, it is a labor-intensive enterprise. The regression results are shown in Table 8, where Columns (3) and (4) correspond to high and low capital intensity, respectively. A significant U-shaped relationship exists between non-state shareholdings and resource misallocation in capital-intensive firms’ mixed-ownership reform, whereas no such pattern exists in labor-intensive firms. Thus, mixed-ownership reform mainly acts on capital-intensive firms through the resource allocation efficiency path, whereas it is more focused on alleviating the redundancy problem in labor-intensive firms, as mentioned in the previous section.

### Regional differences

Significant variation in allocative efficiency exists across regions, and certain location-specific factors heavily impact resource allocation among firms [60]. Mixed ownership reform boosts SOE innovation, and its impact is diverse as it investigates various industries and areas under the influence of the macroeconomic environment. This effect is stronger in monopolistic industries and the developed eastern region [41]. Due to the different levels of overall economic development, industrial structure layout, and talent concentration in different regions, the supply quantity and price marketization of local production factors differ, affecting the resource allocation efficiency of the affiliated enterprises. The eastern, central, and western regions face particularly prominent problems such as unbalanced economic development levels, and a regression is conducted including these regions. The eastern region includes 12 provinces and cities: Beijing, Tianjin, Hebei, Liaoning, Shanghai, Jiangsu, Zhejiang, Fujian, Shandong, Guangdong, Guangxi, and Hainan; the central region includes nine provinces: Shanxi, Jilin, Heilongjiang, Anhui, Jiangxi, Henan, Hubei, Hunan, and Inner Mongolia; and the western region includes 10 provinces and autonomous regions: Chongqing, Sichuan, Guizhou, Yunnan, Tibet, Shaanxi, Gansu, Qinghai, Ningxia, and Xinjiang. We investigate the impact of the depth of mixed-ownership reform on the efficiency of enterprises’ resource allocation in different regions. Table 8 present the results. A significant U-shaped relationship is observed between non-state shareholders’ shareholding and resource misallocation in the eastern region, and the extreme point is within the optimal range of 10%–20%, which passes the subsequent quadratic type test. This relationship is not significant in the mid-west region. This shows that it is feasible to improve the resource allocation efficiency of SOEs in the eastern region using in-depth mixed-ownership reform, whereas the central and western regions still need to build a modern infrastructure system and improve the local market environment. A good market environment is the basis for improving the efficiency of enterprises’ resource allocation through mixed-ownership reform.

### Government resource inclination

Wang et al. (2021) [61] found a significant negative correlation between the intensity ratio of mixed-ownership reform in Chinese enterprises and the degree of tax avoidance from 2003 to 2018. Liu and Shi (2010) [62] propose that the government should maintain and strengthen the monopoly position of large- and medium-sized SOEs by reaping huge monopoly profits, which leads to inefficient resource allocation and loss of social welfare. Improving the soft budget constraint faced by SOEs is an important means of improving the efficiency of resource allocation in China. Local SOE dependence significantly reduces resource allocation efficiency at the city’s industry level. The more severe the local SOE dependency, the more difficult it is for incumbent non-SOEs to obtain resources to expand their production scale, the higher the entry barriers that new entrants face, and the less likely inefficient SOEs are to exit the market [63]. To verify the impact of government resource tilt on SOEs’ mixed-ownership reform, regression analyses were conducted by considering whether the executives of SOEs have official background and whether the enterprises enjoy high government subsidies in the current year, respectively. As shown in Table 9, Columns (1) and (2) correspond to whether the executives of SOEs have an official background or not, respectively. When the enterprises’ executives have an official background, a significant U-shaped relationship exists between non-state shareholders’ shareholding and enterprises’ resource misallocation with an extreme value point of 17.43%, which lies within the optimal interval and passes the subsequent quadratic test. No U-shaped relationship effect is observed for firms whose executives have no official background. This indicates that with the entry of non-state shares, government resource-leaning SOEs are more likely to form goals and behavior congruence with non-state capital at the initial stage, pursuing economic performance goals and improving resource allocation efficiency based on their own social responsibility goals. However, when non-state shareholders’ shareholding exceeds the optimal shareholding, the goal conflict between state-owned and non-state-owned capital becomes more obvious, leading to the deterioration of SOEs’ resource allocation efficiency. If the amount of government subsidy received in the current year is greater than the average amount of government subsidy received by all enterprises in the current year, the enterprise is judged to be highly subsidized; otherwise, it is considered a low-subsidy enterprise. As shown in Table 9, the enterprise in Column (3) is classified as a high-subsidy treatment enterprise, and that in Column (4) is classified as a low-subsidy treatment enterprise. The results show a significant U-shaped relationship between the non-state shareholders’ shareholdings in the hybrid reform and the degree of resource misallocation when enterprises enjoy high government subsidies. As hybridization progresses, the degree of resource misallocation tends to first decrease and then worsen in highly government-subsidized enterprises. However, the same pattern does not exist for enterprises with low government subsidy. The results of the zombie enterprise governance in the SOE reform process are validated. SOEs enjoying high subsidies tend to assume certain social responsibilities and are thus more likely to reach a situation in which their goals and behaviors change from congruent to conflicting with non-state capital in the hybrid reform process. This leads to a U-shaped relationship between non-state shareholders’ shareholding and the degree of SOEs’ resource misallocation.

**Table 9:** Expansive analysis: Government resource tilt and external market environment.

*MA*	(1)	(2)	(3)	(4)	(5)	(6)
	With official background	No official background	High Subsidy Treatment	Low Subsidy Treatment	High level of marketability	Low level of marketability
*nonshr*	-34.32**	-7.064	-17.29**	3.227	-20.47*	-1.612
	-15.42	-11.25	-8.695	-153.3	-11.12	-14.75
*nonshr* ^2^	92.22***	31.12	47.08**	67.79	54.32**	0.654
	-32.62	-27.61	-19.38	-542.4	-24.08	-38.9
*Controlvariables*	Controlled	Controlled	Controlled	Controlled	Controlled	Controlled
*Year*	Controlled	Controlled	Controlled	Controlled	Controlled	Controlled
*Firm*	Controlled	Controlled	Controlled	Controlled	Controlled	Controlled
_*cons*	-52.52**	-40.5	-41.53***	-134.8	-45.68**	-130.8**
	-25.54	-39.09	-16.09	-377.3	-20.27	-57.42
*Adj* − *R*^2^	0.045	0.037	0.033	0.16	0.039	0.047
*N*	2425	4334	6434	325	5248	1511

T-values are in parentheses;

***, **, and * indicate statistical significance at the 1%, 5%, and 10% levels, respectively.

### Differences in the external institutional environment

Due to the different external institutional environments, enterprises face different degrees of marketization and differences in the prices and supply of local production factors, which affect the efficiency of enterprises’ resource allocation. Since 2016 is used as the base period in the China Marketization Index by Province Report (2021), the 2016–2019 marketization index cannot be directly compared with the 2008–2016 data and scores; therefore, the China Marketization Index by Province Report (2018) is still used, where the 2017–2018 data are extrapolated from the 2008–2016 data [64]. When the marketization index of the enterprise’s location is greater than the median marketization index of all regions in that year, mkt takes the value of 1, indicating that the enterprise is in a high-marketization region; otherwise, it is 0, indicating that the enterprise is in a low-marketization region. Columns (5) and (6) in Table 9 correspond to the high and low marketization process regions, respectively. The regression results show a significant U-shaped relationship between non-state shareholders’ shareholding and resource misallocation in regions with high marketization process, whereas no such relationship is observed in low marketization regions. Regions with high marketization process have fewer resource constraints and less restrictions on the free flow of production factors, and SOEs are better able to achieve improved resource allocation efficiency through deeper hybrid reform. To a certain extent, the effectiveness of SOE reforms is closely related to market-oriented reforms. While promoting mixed-ownership reform, attention should also be paid to optimizing the market environment.

### The counterfactual enhancement effect of resource misallocation in mixed-ownership reform

A diverse range of mixed shareholders, higher level of mixed equity, and fewer limitations on mixed equity have a significant positive impact on promoting green innovation among SOEs [65]. SOEs are often more innovative than non-state-owned enterprises, and an increase in SOE shareholding enhances the innovation capabilities of mixed-ownership enterprises, for which the level of national shareholding inversely affects the reliance on organizational control capabilities for innovation in corporate governance [48]. This study reveals a significant U-shaped relationship between the shareholding of non-state shareholders and the degree of SOEs’ resource misallocation. When mixed-ownership reform has been implemented to a certain extent, and the shareholding of non-state shareholders reaches the optimal shareholding amount, SOEs’ resource allocation efficiency can be optimized regardless of other external factors. How much room does mixed-ownership reform leave to improve resource allocation efficiency? Assuming that the non-state shareholders of all sample SOEs achieve an optimal shareholding of 17.95%, a counterfactual prediction is made using the regression equation of Model (1). It is found that when SOEs achieve the optimal degree of hybridization, the median degree of resource misallocation decreases from 0.8821 to 0.0915(an improvement of 89.6%), indicating that deepening mixed-ownership reform is an effective way to improve SOEs’ resource allocation efficiency on average.

## Conclusion

To evaluate whether the reform of SOEs will achieve results, most studies have theoretically explored the reasons for the inefficiency of SOEs and then further analyzed the changes in reformed enterprises to verify the reasons that affect their efficiency. In the current economic operation, due to various factors, the factor market is distorted, and resources are misallocated. According to the Solow model and its decomposition, economic growth comes from the input of production factors, technological progress, and resource allocation efficiency. Enterprises’ resource input must incur more costs, and improving resource allocation efficiency is an important aspect of future economic development. This study drew on the HK model and established a framework for measuring the impact of mixed-ownership reform on SOEs’ resource allocation efficiency based on Zhang and Deng (2020) [28], providing a microscopic research perspective on the effectiveness of SOEs’ mixed-ownership reform. It calculated the industrial added value of SOEs and the degree of resource misallocation according to the income method announced by the National Bureau of Statistics using the annual report data of A-share listed companies from 2008 to 2018. Using the CSMAR database, combined with information from Tianyancha, Qichacha, and the company’s official website, the nature of SOEs’ top 10 shareholders was manually calculated, and the impact of mixed-ownership reform on SOEs’ resource allocation efficiency was empirically analyzed.

It was found that the depth of mixed-ownership reform has an important impact on the efficiency of resource allocation of SOEs, and 80% of SOEs listed as A-shares from 2008 to 2018 have introduced non-state shareholders’ shareholding to varying degrees to implement mixed-ownership reform. The “country into the people” retreat cannot achieve the optimal efficiency of SOEs’ resource allocation; moreover, the top 10 shareholders in the proportion of private and foreign shares is not the more the better, nor the less the better, but an optimal interval. With the entry of non-state-owned shareholders, the U-shaped relationship between the degree of mixed-ownership reform and the degree of SOEs’ resource misallocation first decreases and then increases. However, only 10% of the enterprise sample reached the optimal range of non-state shareholding during the mixed-ownership reform process. Further analysis revealed a significant U-shaped relationship between the shareholding of non-state shareholders and the distortion of capital factor input, which first inhibits and then worsens the distortion of capital input. Nevertheless, a non-significant inverted U relationship with the distortion of labor factor input is observed, which is consistent with the previous literature showing that mixed-ownership reform can alleviate the policy burden of SOEs. Simultaneously, a significant U-shaped relationship is formed between the shareholding of non-state shareholders and the degree of SOE resource misallocation through the combined effect of financing cost benefits and labor input distortion constraints.

The impact of non-state shareholders’ shareholding on SOEs’ resource allocation efficiency is highly heterogeneous. The U-shaped relationship presented has obvious differences in different industries (competitive industry, capital-intensive industry, etc.), the location, the degree of government resource inclination, and different external market environment. For eastern enterprises with competitive and high capital intensity, those enjoying a favorable treatment of government resources, and those with a good external market environment, the effect of the depth of mixed-ownership reform on resource allocation efficiency improves and may deteriorate later.

SOEs implement mixed-ownership reform to optimize the allocation of resource elements, promote a reasonable flow of elements, influence the scale of enterprises and narrow the gap between their actual scale and their ideal scale, and give better play to the scale effect of enterprises. The shareholding of non-state shareholders should be kept in the optimal range to further deepen the reform of SOEs through the introduction of non-state shareholders for mixed-ownership reform and improve the efficiency of enterprises’ resource allocation to promote high-quality economic development. Simultaneously, policy objectives should be moderately adjusted to break the segmentation of factor markets into different regions and improve the distortion of factor inputs. To enhance SOEs’ economic efficiency, non-state shareholders are introduced to participate in enterprise decision-making and corporate governance. The efficiency of resource allocation under existing constraints can be improved by alleviating the insufficient input of capital factors and the excessive input of labor factors.

Due to SOEs’ different functional positions, a classification reform is implemented. The moderate mixed-ownership reform of competitive SOEs can improve economic efficiency. To achieve high-quality economic development, local governments should further improve the local business environment and promote the free flow of production factors.

In SOEs’ mixed-ownership reform, it is necessary to pay more attention to the corporate governance capabilities and social responsibility of non-state-owned capital to improve the consistency of goals between non-state-owned capital and state-owned capital and make better use of mixed-ownership reform to improve the efficiency of SOEs’ resource allocation.

This article primarily focuses on the relationship between ownership structure and the efficiency of resource allocation in state-owned enterprises. In future studies, the research scope can be broadened. Digital infrastructure plays a crucial role in facilitating the digital transformation of businesses, particularly in significantly benefiting the digital transformation of privately owned enterprises [66]. Financial resources can lead to industrial prosperity, improvements in education, and technological advancements [67]. Therefore, digital resources and financial resources can be integrated into the resource allocation efficiency framework of state-owned enterprises for research. In addition, future research could explore the integration of resource allocation efficiency and innovation performance in state-owned enterprises. Wu et al. (2023) [68] examined the knowledge performance of enterprise incubators from the perspective of gatekeepers. On the one hand, the innovation performance of state-owned enterprises is reflected in the innovation of their own products and services. On the other hand, it is also reflected in the spillover effect of innovation, which fosters the development of more innovative enterprises and enhances the overall innovation level of the region.

## Supporting information

S1 Data (XLSX)

## References

[1] Restuccia D, Rogerson R. Misallocation and productivity. Review of Economic dynamics. 2013 Jan;16(1):1–10. doi: 10.1016/j.red.2012.11.003

[2] Midrigan V, Xu DY. Finance and misallocation: Evidence from plant-level data. American economic review. 2014 Feb;104(2):422–458. doi: 10.1257/aer.104.2.422

[3] Bai P, Cheng W. Labour misallocation in China: 1980–2010. Applied Economics. 2015 Dec;48(25):2321–2332. doi: 10.1080/00036846.2015.1119790

[4] Andritzky J, Kassner B, Reuter WH. Propagation of changes in demand through international trade: A case study of China. The World Economy. 2018 Aug;42(4):1259–1285. doi: 10.1111/twec.12725

[5] Linnenluecke MK, Marrone M, Singh AK. Sixty years of Accounting & Finance: a bibliometric analysis of major research themes and contributions. Accounting & Finance. 2020 Nov;60(4):3217–3251. doi: 10.1111/acfi.12714

[6] Ngwenya N, Simatele MD. Unbundling of the green bond market in the economic hubs of Africa: Case study of Kenya, Nigeria and South Africa. Development Southern Africa. 2020 Feb;37(6):888–903. doi: 10.1080/0376835X.2020.1725446

[7] Tu Z, Xiao G. The industry characteristics of total factor productivity growth of large and medium-sized industrial enterprises during the transition period. Statistics and Decision. 2005 Dec;23:64–65.

[8] Hsieh CT, Klenow PJ. Misallocation and manufacturing TFP in China and India. The Quarterly journal of economics. 2009 Nov;124(4):1403–1448. doi: 10.1162/qjec.2009.124.4.1403

[9] Wu X. Analysis of environmental governance expense prediction reform with the background of artificial intelligence. Journal of Organizational and End User Computing (JOEUC). 2015;34(5):1–19. doi: 10.4018/JOEUC.287874

[10] Gopinath G, Kalemli-Özcan Ş, Karabarbounis L, Villegas-Sanchez C. Capital allocation and productivity in South Europe. Quarterly Journal of Economics. 2017 Jun;132(4):1915–1967. doi: 10.1093/qje/qjx024

[11] Nie H, Jia R. The productivity of manufacturing firms and misallocationsn in china. World Economy. 2011 Nov;7:23–34.

[12] Acemoglu D, Laibson D, List JA. Aggregate incomes. London: Pearson Education Limited; 2016.

[13] He W, Zhang Y, Zhong Y, Chen J. The impact of income gap on the inverted U-shaped total factor productivity and its mechanisms: Evidence from transnational-level analysis. Plos one. 2020 Jan;15(1):e0228023. doi: 10.1371/journal.pone.0228023 31990960 PMC6986713

[14] Gai Q, Zhu X, Shi Q. Labor market’s distortion, structural change and labor productivity in China. Economic Research Journal. 2013 May;5:87–97.

[15] Yuan Z, Xie D. The effect of labor misallocation on TFP: China’s evidence 1978–2007. Economic Research Journal. 2011 Jul;7:4–17.

[16] Wang L, Yuan L. Was capital misallocation an important factor for loss in total factor productivity?. Statistical Research. 2014 Aug;31(8):11–18.

[17] Zhang P, Ma H. Borrowing constraints and misallocations: Empirical evidence from China. Journal of Tsinghua University Science and Technology. 2012 Sep;52(9):1303–1308.

[18] Bin P, Chen X, Fracasso A, Tomasi C. Resource allocation and productivity across provinces in China. International Review of Economics & Finance. 2018 Feb;57:103–113. doi: 10.1016/j.iref.2018.02.015

[19] Sun W, Guo Y. Unequal China: The political economy and cultural politics of inequality. London: Routledge. 2013.

[20] Chen J, Chen J, Miao Y, Song M, Fan Y. Unbalanced development of inter-provincial high-grade highway in China: Decomposing the Gini coefficient. Transportation Research Part D: Transport and Environment. 2016 Oct;48:499–510. doi: 10.1016/j.trd.2015.06.008

[21] Xiong N, Wong SW, Ren Y, Shen L. Regional disparity in urbanizing china: Empirical study of unbalanced development phenomenon of towns in southwest China. Journal of Urban Planning and Development. 2020;146(3):05020013. doi: 10.1061/(ASCE)UP.1943-5444.0000586

[22] Zhang S, Chen C, Xu S, Xu B. Measurement of capital allocation efficiency in emerging economies: Evidence from China. Technological Forecasting and Social Change. 2021 Oct;171:120954. doi: 10.1016/j.techfore.2021.120954

[23] Wang J, Cheng-Han T. Mixed Ownership Reform and Corporate Governance in China’s State-Owned Enterprises. Vanderbilt Journal of Transnational Law. 2020 Jan;53:1055–1089.

[24] Li S, Xia J. The roles and performance of state firms and non-state firms in China’s economic transition. World Development. 2008 Jun;36(1):39–54. doi: 10.1016/j.worlddev.2007.01.008

[25] Liu X, Li L. The impacts of gaizhi on enterprises performance in Chinese industry. China Industrial Economics. 2005 May;3:5–12.

[26] Yin Z, Liu L, Wang H, Wen F. Study on the ownership balance and the efficiency of mixed ownership enterprises from the perspective of heterogeneous shareholders. PloS one. 2018 Apr;13(4):e0194433. doi: 10.1371/journal.pone.0194433 29614126 PMC5882114

[27] Shleifer A, Vishny RW. A survey of corporate governance. Journal of Finance. 1997;52(2):737–783. doi: 10.1111/j.1540-6261.1997.tb04820.x

[28] Zhang T, Deng Y. Development zone, resource allocation and macroeconomic efficiency-empirical research based on Chinese industrial enterprises. China Economic Quarterly. 2020 May;19(4):1237–1266.

[29] Chu SN, Song L. Promoting private entrepreneurship for deepening market reform in China: A resource allocation perspective. China & World Economy. 2015 Jan;23(1):47–77. doi: 10.1111/cwe.12099

[30] Jiang T, Sun K, Nie H. Administrative rank, total factor productivity and resource misallocation in Chinese cities. Management World. 2018 May;34(3):38–50+77+183.

[31] Melitz MJ, Polanec S. Dynamic Olley-Pakes productivity decomposition with entry and exit. The Rand journal of economics. 2015 Apr;46(2):362–375. doi: 10.1111/1756-2171.12088

[32] Hsieh CT, Hurst E, Jones CI, Klenow PJ. The allocation of talent and us economic growth. Econometrica. 2019 Sep;87(5):1439–1474. doi: 10.3982/ECTA11427

[33] Dias DA, Marques CR, Richmond C. Misallocation and productivity in the lead up to the Eurozone crisis Journal of Macroeconomics. 2016 Sep;49:46–70. doi: 10.1016/j.jmacro.2016.04.009

[34] Oberfield E. Productivity and misallocation during a crisis: Evidence from the Chilean crisis of 1982. Review of Economic Dynamics. 2013 Jan;16(1):100–119. doi: 10.1016/j.red.2012.10.005

[35] Brandt L, Tombe T, Zhu X. Factor market distortions across time, space and sectors in China. Review of Economic Dynamics. 2013 Jan;16(1):39–58. doi: 10.1016/j.red.2012.10.002

[36] Dollar D, Wei SJ. Das (Wasted) Kapital: Firm Ownership and Investment Efficiency in China. IMF Working Papers. 2007 Jan;009.

[37] Song Z, Storesletten K, Zilibotti F. Growing like china. American economic review. 2011 Feb;101(1):196–233. doi: 10.1257/aer.101.1.196

[38] Zhang S, Luo J, Huang DH, Xu J. Market distortion, factor misallocation, and efficiency loss in manufacturing enterprises. Journal of Business Research. 2023 Jan;154:113290. doi: 10.1016/j.jbusres.2022.08.054

[39] Lin KJ, Lu X, Zhang J, Zheng Y. State-owned enterprises in China: A review of 40 years of research and practice. China Journal of Accounting Research. 2020 Mar;13(1):31–55. doi: 10.1016/j.cjar.2019.12.001

[40] Yuan R, Li C, Sun X, Khaliq N. Mixed-ownership reform and strategic choice of Chinese state-owned enterprises. Plos one. 2023 Apr;18(4):e0284722. doi: 10.1371/journal.pone.0284722 37083868 PMC10121011

[41] Zhang X, Yu M, Chen G. Does mixed-ownership reform improve SOEs’ innovation? Evidence from state ownership. China Economic Review. 2020;61:101450. doi: 10.1016/j.chieco.2020.101450

[42] Zhang S, Zhang W, Chen F, Guo B. Does the mixed-ownership reform of Chinese state-owned enterprises improves their total factor productivity?. Pacific-Basin Finance Journal. 2023 Dec;82:102182. doi: 10.1016/j.pacfin.2023.102182

[43] Li J. Does the mixed ownership reform of state-owned enterprises enhance investment efficiency?. Business and Management Journal. 2021 Feb;43(2):56–70.

[44] Lisheng HE, Renqin G. Study on the mixed ownership economy in China. Journal of Nanjing Uni-versity of Finance and Economics. 2020 Arp;4:44–47.

[45] Wu L, Li J, Qi J, Shi N, Zhu H. How to promote public engagement and enhance sentiment through government social media during the COVID-19 crisis: A public value management perspective. ournal of Organizational and End User Computing (JOEUC). 2022;34(6):1–24. doi: 10.4018/JOEUC.308819

[46] Huang J. Incentive selection and privatization of managers in joint-stock state-owned enterprises: Application of mixed oligopoly model. World Economic Papers. 2005 Feb;2:42–50.

[47] Ma L, Wang L, Zhang Q. Pecking order of mixed ownership: The logic of market. China Industrial Economics. 2015 Jul;7:5–20.

[48] Lo D, Gao L, Lin Y. State ownership and innovations: Lessons from the mixed-ownership reforms of China’s listed companies. Structural Change and Economic Dynamics. 2022 Mar;60:302–314. doi: 10.1016/j.strueco.2021.12.002

[49] Zhou KZ, Gao GY, Zhao H. State ownership and firm innovation in China: An integrated view of institutional and efficiency logics. Administrative Science Quarterly. 2016 Oct;62(2):375–404. doi: 10.1177/0001839216674457

[50] Cai G, Zheng G, Ma X, Lu R. Decentralization and mixed-ownership reform in China. Economic Research Journal. 2018 Sep;53(9):99–115.

[51] Broda C, Weinstein DE. Globalization and the Gains from Variety. The Quarterly journal of economics. 2006 May;121(2):541–585. doi: 10.1162/qjec.2006.121.2.541

[52] Hendel I, Nevo A. Measuring the Implications of Consumer Inventory Behavior. Econometrica. 2006 Nov;74(6):1637–1673. doi: 10.1111/j.1468-0262.2006.00721.x

[53] Hao Y, Gong L. State and private non-controlling shareholders in SOEs and private firms, and firm performance. Economic Research Journal. 2017 Mar;3:122–135.

[54] Li Y. Several misunderstandings about mixed ownership must be clarified. Xian Feng Dui. 2014 Nov;11:8–9.

[55] Zeng Q, Chen X. State stockholder, excessive employment and labor cost. Economic Research Journal. 2006 May;5:74–86.

[56] Yang M, Yang F, Sun C. Factor market distortion correction, resource reallocation and potential productivity gains: An empirical study on China’s heavy industry sector. Energy Economics. 2018 Jan;69:270–279. doi: 10.1016/j.eneco.2017.11.021

[57] Aharony J, Lee CWJ, Wong TJ. Financial packaging of IPO firms in China. Journal of Accounting Research. 2000;38(1):103–126. doi: 10.2307/2672924

[58] Zhao J, Wang Y, Jin P, Yang C. The optimal managerial incentive mechanism for China’s local and central SOEs: An empirical study of listed companies. Corporate Board: role, duties and composition. 2017;13:79–86.

[59] Guan J, Gao Z, Tan J, Sun W, Shi F. Does the mixed ownership reform work? Influence of board chair on performance of state-owned enterprises. Journal of Business Research. 2021 Jun;122:51–59. doi: 10.1016/j.jbusres.2020.08.038

[60] Bin P, Chen X, Fracasso A, Tomasi C. Resource allocation and productivity across provinces in China. International Review of Economics & Finance. 2018 Sep;57:103–113. doi: 10.1016/j.iref.2018.02.015

[61] Wang W, Wang H, Wu JG. Mixed ownership reform and corporate tax avoidance: Evidence of Chinese listed firms. Pacific-Basin Finance Journal. 2021 Oct;69:101648. doi: 10.1016/j.pacfin.2021.101648

[62] Liu R, Shi L. The dual efficiency loss of state-owned enterprises and economic growth. Economic Research Journal. 2010 Jan;1:127–137.

[63] Yan L, Rudai Y. Dependence of local state-owned enterprises, improvement of resource allocation efficiency and supply side Reform. Economic Research Journal. 2018 Feb;53(605):82–96.

[64] Wang X, Fan G, Hu L. Marketization Index of China’s Provinces: Neri Report 2018. Social Sciences Academic Press (CHINA): Beijing. 2019 Feb;39–61.

[65] Yuan R, Li C, Li N, Khan MA, Sun X, Khaliq N. Can mixed-ownership reform drive the green transformation of SOEs?. Energies. 2021 Apr;14(10):2964. doi: 10.3390/en14102964

[66] Wu W, Wang S, Jiang X, Zhou J. Regional digital infrastructure, enterprise digital transformation and entrepreneurial orientation: Empirical evidence based on the broadband china strategy. Information Processing & Management. 2023 Sep;60(5):103419. doi: 10.1016/j.ipm.2023.103419

[67] Xia Ds, Kong CL. The Impact of Digital Inclusive Finance on Rural Revitalization: Evidence From China. Journal of Organizational and End User Computing. 2024;36(1):1–18. doi: 10.4018/JOEUC.337970

[68] Wu W, Zhai M, Liu J, Huang X. Gatekeeper Networks, Knowledge Inertia, and Knowledge Performance in Business Incubators. Journal of Organizational and End User Computing. 2023;35(1):1–33. doi: 10.4018/JOEUC.333634

